# Adolescents’ perspectives on everyday life with obesity: a qualitative study

**DOI:** 10.1080/17482631.2018.1479581

**Published:** 2018-06-18

**Authors:** Gudbjørg Øen, Bente Kvilhaugsvik, Kari Eldal, Anne-Grethe Halding

**Affiliations:** a Faculty of Health and Social sciences, Western Norway University of Applied Sciences, Bergen, Norway; b Faculty of Health and Social sciences, Western Norway University of Applied Sciences, Stord, Norway; c Faculty of Health and Social sciences, Western Norway University of Applied Sciences, Førde, Norway

**Keywords:** Adolescents, children, obesity, experience, shame, social support, communication, qualitative

## Abstract

**Purpose:** This study aimed to gain an in-depth understanding of the perspectives and life experiences of adolescents living with obesity. **Methods:** Five adolescents living with obesity were involved in repeated interviews, and qualitative content analysis was performed. **Results:** Three themes emerged: obesity as a multi-faceted and difficult to solve condition; obesity as a shameful and vulnerable subject; and bullying and fragile social relationships. Adolescents living with obesity described everyday life challenges as difficult to interpret and solve. Adolescents living with obesity perceived causes for obesity differently, and those who emphasised familial determinants seemed to face greater challenges. Lack of support from parents, trusted friends and health-care providers and bullying, shame, guilt and self-blame represented threats that decreased motivation for help seeking and successful life-style changes. The adolescents were ambivalent regarding disclosing their concerns and seeking help. The adolescents feared that health care providers would demand too much from them, and peers were perceived as a possible source of support. **Conclusion:** Care providers need to be skilled in assessing each individual’s resources and interpretations of their condition, to be able to communicate in a respectful, patient-centred manner and to assist adolescents to explore their ambivalence and set their own realistic goals. More research is needed.

## Introduction

Childhood obesity is one of the most serious public health challenges of the twenty-first century, with figures increasing at an alarming rate. According to the Norwegian Institute of Public Health (NIPH), there has been a steady increase in the number of overweight children in Norway over the past 30 years. The prevalence of children living with overweight, including those classified as obese, in a sample of third-grade Norwegian children (mean age 8,3 years) was 19% and 4% respectively (Biehl et al., 2013).

Childhood and adolescent obesity raise concerns with respect to potential physical and psychosocial consequences. Children and adolescents living with obesity are likely to remain obese into adulthood and to develop non-communicable diseases such as diabetes and cardiovascular diseases at a young age. There is also an increase in social health disparities (Loring & Robertson, 2014). Obesity is associated with decreased quality of life, low self-esteem, bullying, with somatic pain, reduced physical activity and an increase in sedentary behaviour. There is also a correlation between childhood obesity and the parents´ educational level and weight status (Buttitta, Iliescu, Rousseau, & Guerrien, 2014). Increased weight status in children is associated with poorer educational outcomes (Carey, Singh, Brown, & Wilkinson, 2015).

Obesity is a multifactorial condition, subject to different interpretations. According to Lachal et al. (2013), three groups—children and adolescents, parents and health professionals—experienced the same difficulty in perceiving and labelling obesity primarily because of their lack of any real common ground. Insufficient shared understanding, such as described above, has the potential to destabilize the therapeutic relationship. This incongruence might be a result of health-care providers’ failing to understand what families can realistically achieve, and equally, families may have unrealistic expectations in terms of the outcome of interventions (Staniford, Breckon, Copeland, & Hutchison, 2011). Hence, there is a need for these different parties to communicate their understanding and perspective on obesity with each other. By taking on board the personal experiences of children and adolescents living with obesity, health workers may expand their understanding of obesity to match treatment plans to the needs and expectations of these user populations (Chung, Sherman, Goodman, Bickham, & Rich, 2013; Lachal et al., 2013; Øen & Stormark, 2013).

Adolescents’ weight experiences in varied contexts have been described. Children living with obesity emphasized the social impact of body size, describing experiences of abuse and isolation. They actively assessed their own size; many wished their bodies were different, and some were anxious about their shape (Rees et al., 2011). Some experienced stigma, discrimination and humiliation from health-care professionals because of their weight and an increasing culture of blame towards people living with obesity (Thomas, Hyde, Karunaratne, Herbert, & Komesaroff, 2008). In an obesity treatment programme, health-related text messages from health-care providers to adolescents had the potential to heighten their sense of shame (Smith, Kerr et al., 2014). Body size was perceived as being under the individual’s control (Rees et al., 2011), and children took ultimate responsibility for their weight status and expressed significant feelings of guilt and shame (Kirk et al., 2014) but denied any link between responsibility and guilt (Braun, Schell, Siegfried, Müller, & Ried, 2014). The stigma associated with being overweight or obese was identified as a major barrier and potentially prevented adolescents from looking for help (Smith, Straker, McManus, & Fenner, 2014). Some tried to lose weight and had suggestions for action (Smith, Kerr, Fenner, Straker, 2014; Rees, Caird, Dickson, Vigurs, & Thomas, 2014; Wills, Backett-Milburn, Gregory, & Lawton, 2006), but some experienced lack of motivation and barriers to change and disliked being compared with other adolescents with obesity (Smith, Kerr, Fenner, Straker, 2014). Obesity was also framed as a problem primarily located within the family, not in the wider environment. The family was identified as being the primary and most important instigator of preventive measures (Braun et al., 2014). Adolescents living with obesity perceived that attempts to change eating-practice had to be negotiated with friends and family (Wills et al., 2006). Friends and family also often played a major role in motivating and supporting them (Woolford et al., 2012). Adolescents rarely mentioned any health-related consequences of their own and others’ fatness, although wearing “nice” clothes and being “slowed down” were flagged as considerations by girls and boys, respectively. Being very obese led to anxiety, and subjects described attempts at “crash dieting” (Wills et al., 2006). Adolescents living with obesity hated or disliked the word “obesity,” would rather be called “fat” or “overweight” (Thomas et al., 2008) and had difficulty in perceiving and labelling obesity (Lachal et al., 2013).

To sum up, these studies describe how adolescents living with obesity experience strain in everyday life, emphasizing the negative psychosocial consequences and sometimes contradictory meaning of obesity. However, children and adolescents living with obesity were rarely engaged in discussion of interventions, and few study findings had depth or breadth (Rees et al., 2011). A systematic review describes adolescent obesity as a “neglected topic” and notes that the lack of clinical guidance and expert opinion makes this a priority research area given the high prevalence of adolescent obesity and its significant longer-term health impacts (Shrewsbury, Baur, Nguyen, & Steinbeck, 2014). We have not been able to identify any studies from Norway describing the life experiences of adolescents living with obesity. Health-care professionals in Norway have become highly concerned about the increasing number of obese children and adolescents. The “Healthy Future” (HF) project originating in western Norway aims to seek knowledge of how evidence-based interventions could be developed and implemented in this specific area of primary health care (Øen & Stormark, 2013). Every step to reduce overweight has to be taken by the patient himself/herself, tailored to the environment in which he or she lives. To develop effective interventions and prepare implementation, the subject’s perspective has to be considered.

### Aims

This study aimed to gain an in-depth understanding of the perspectives and life experiences of adolescents living with obesity to guide interventions and strengthen the patient-clinician partnership. In this paper, we answer the following research questions:

– How do adolescents experience everyday life with obesity?
– How do adolescents living with obesity make sense of their condition?
– How do adolescents living with obesity describe their challenges and motivation for changing behaviour?
– How do adolescents living with obesity experience health-care encounters?

## Methods

### Design

We chose a descriptive, qualitative approach with individual qualitative interviews and qualitative content analysis, following Graneheim and Lundman (2004) and Graneheim, Lindgren, and Lundman (2017), to explore the meaning of adolescents’ perspectives on everyday life with obesity. Qualitative content analysis focuses on the subjects’ experiences and reflections, emphasizing context and variation. This method offers a systematic interpretive approach by analysing both manifest and latent meaning content of transcribed interview text, based on the assumptions that a qualitative analysis is always an interpretation and the text involves multiple meanings. Interviewers and interviewees create data mutually, and the interpretation continues during the researchers’ analysis of the text written later (Graneheim et al., 2017).

### Participants

The study included obese adolescents (>30-isoBMI) 12–15 years old who had, in some way, been in contact with health-care providers about their obesity. Adolescents who did not understand the Norwegian language were excluded. This age group was chosen because guidelines from the Norwegian Health Directorate require mandatory measurement of height and weight in adolescents in the eighth grade. We planned a purposive sampling to choose cases illustrating the specific topic in which we were interested (Silverman, 2005). The recruitment was initiated by contacting school health nurses, who carried out the mandatory measurement in 11 surrounding municipalities. However, few participants were recruited this way, and, thus, we advertised the study in a local newspaper. A newspaper article reporting the HF project recruited the first participant, three participants from three municipalities were recruited by school health nurses, and one participant recruited an obese friend. Between November 2013 to February 2014, we conducted 10 individual interviews with five adolescents 12–15 years old. The sample comprised three girls and one boy in the eighth grade and one girl in the ninth grade. They were all Norwegians citizens, living with their parents. Two of the adolescents lived in double-households with divorced parents.

### Ethical considerations

For all of the participants, an informed consent was obtained from both the adolescent and the parent, following presentation of written information. The study was presented to the Regional Committee for Medical and Health Research Ethics (REC), and the project (NO: 29263) was approved by The Norwegian Social Science Data Services (NSD).

### Data collection

Individual in-depth interviews were carried out based on a semi-structured interview guide. (Table I). The first author (GØ) interviewed four participants, and the second author (BK) interviewed one participant. The study was planned with two interviewers to minimize the impact of each interviewer’s pre-understanding and increase the study’s credibility through the interview process and further analysis (Rowan & Huston, 1997). Both interviewers were female nurses experienced in health-promoting conversations with adolescents and qualitative research interviews. The interviews occurred in the school health nurses’ offices for four of the adolescents and in the first author’s office for one adolescent, according to each youngster’s preference. Each interview lasted 40–85 minutes, was audiotaped, and transcribed verbatim by the second author (BK). We focused on carrying out an empathic, accepting and client-centred conversation using open-ended questions, offering support when needed. After we carried out preliminary analysis of five interviews and were unable to recruit more participants, we decided to carry out a more in-depth exploration of some of the data by interviewing each participant a second time to ensure that we grasped the meanings and nuances. The same interviewers met the participants for the second interview. The time lag between the two interviews was five to 8 weeks. Ahead of the second interviews, the participants were presented with a summary of the first interview, and we offered the opportunity, with ample time, to make corrections or additions. To reach a best possible shared interpretation of their meaning, we also used open questions to explore what each youth expressed in the first interviews. The interviewing continued until no new significant information was produced.

**Table I.** Interview guide.

Main questions asked in the individual interviews with adolescents with obesity
Could you please share your experiences being bigger than the normal?
What is your opinion on possible causes of obesity?
What do you believe could decrease your obesity?
Could you describe if you have experienced trying to reduce your weight? What occurred? What was the outcome for you?
Could you please share your experiences of getting help from anyone in the process of losing weight, and what was the outcome for you?
How do you experience health-care encounters?

### Data analysis

Each interview was the unit of analysis for qualitative content analysis with a search for meanings (Graneheim et al., 2017; Graneheim & Lundman, 2004). The third author (KE) imported the data into QSR NVivo (Qualitative Solutions and Research International) and sorted the text into suggested meaning units and abstraction into data-developed codes and categories. All of the authors then read all of the transcripts and provided additional suggestions for codes and categories. During a group meeting, with all of the authors present, we compared and agreed on final categories and subcategories (the manifest content), and through further reflexive dialogues in subsequent meetings, we reached consensus on the latent meaning of the text and the themes produced within the data. We based all of the codes and categories on the phenomena raised by the participants, and the meaning content and themes were developed within the frame of the study’s aim and research questions. The life experience of being obese, making sense of the situation, efforts to manage the situation and experience of health-care encounters, as well as the social and cultural manifestations of these issues, were emphasized during the analysis. We present an example of the development from meaning units through codes and categories to themes in Table II to illustrate the trustworthiness of the analysis.

**Table II.** Example of the development from meaning units, through codes and categories, to themes in the qualitative content analysis.

Theme	Main category	Category	Sub-category	Code	Meaning unit
Obesity as a multi-faceted and “difficult—to-solve” condition	Being overweight or obese	Causal explana-tions	Environmental causes	Food Inactivity	I do not eat pizza very often, but I could do better at avoiding it It might just take small changes, such as walking slightly faster for example.
			Biological causes	Heredity Metabolism	They may have a family history of obesity If I have it, but my father and nana have slow metabolisms
			Psychological causes	Eating because of sad feelings Feeling sad	In a way I have been sad, I have—such as—comfort eaten, because I have felt sad that my grandmother died. I feel I have a tough time. I feel such as I’m excluded
Theme	Main category	Category	Sub-category	Code	Meaning unit
Obesity as a multi-faceted and “difficult—to-solve” condition	Being overweight or obese	Causal explana-tions	Environmental causes	Food Inactivity	I do not eat pizza very often, but I could do better at avoiding it It might just take small changes, such as walking slightly faster for example.
			Biological causes	Heredity Metabolism	They may have a family history of obesity If I have it, but my father and nana have slow metabolisms
			Psychological causes	Eating because of sad feelings Feeling sad	In a way I have been sad, I have—such as—comfort eaten, because I have felt sad that my grandmother died. I feel I have a tough time. I feel such as I’m excluded

### Results

The adolescents expressed gratitude for the interviewers’ interest in their situation, and

the interviewees talked willingly about their life experiences, thus appearing to show trust in the interviewers. However, it seemed unusual for the participants to be asked about their experiences. It might have been challenging to put experiences into words. Sometimes the youngsters expressed sadness or signs of feeling uncomfortable. Using awareness of the subjects’ body language and expressions, we then emphasized supportive communication. Across the 10 interviews, three themes and seven subthemes emerged.

Obesity as a multi-faceted and “difficult-to-solve condition”

- Adolescents’ perspectives on causes of obesity
- Challenges in changing behaviour

Bullying and fragile social relationships

- The important but deceitful friends
- The family as the primary source of support

Obesity as a shameful and sensitive issue

- An urge for secrecy
- The ambiguity of asking for help
- The complex health-care encounters

### Obesity as a multi-faceted and “difficult-to-solve” condition

All of the adolescents were aware of their obesity, had reflected on their situation, and had opinions on why they were obese. The way the participants made sense of their situation seemed to influence their feelings and ways of coping with their obesity.

#### Adolescents’ perspectives on causes of obesity

The participants expressed different views on the possible causes of obesity. Some thought that the condition resulted from eating too much and choosing unhealthy food, sweets or soft drinks or resulted from a lack of physical activity. They were all aware of their weight-problem and own responsibility. One of the participants put it in this way:

I think the situation is mostly down to me. (U3)

In some cases, the family’s eating habits could put an extra burden on adolescents by contributing to their eating habits and obesity. One adolescent expressed it in this way:

I knew I was bigger than I should be. In the country my mum comes from there is so much good food. They use more fat and sugar in the food than they do in Norway, so it’s easy to put on weight because of the food. I find it easy to eat more than I should, and I eat more sweets than I should too, which means that I get bigger too. (U1)

Others felt that genes could cause their condition, a condition that was unfair and difficult to solve. The following quotes illustrate this feeling of a unfair condition:

They (the obese adolescents) may have a family history of obesity, and consequently may be unaware of the situation. In my family, my grandfather, my uncle and my mum are obese. I just got it because of heredity. My sister also has it, but she has been eating…. But I, well I inherited it… She has always had, well—a good appetite (smiles), but I have been such as this since I was born. (U4)

My mom is obese, but she had an operation to lose weight. My sister is also obese, and my stepfather. In addition, then, both my grandparents on my mom’s side, and my aunt, but no one on my dad’s side. (U5)

Thus, heredity and family seemed to trap them and put upon them a situation out of their control, as this participant expressed:

We do not eat many sweets either…we do not really…I do not know what to do about it (looking sad, speaking quietly). We have never had chocolate spread at home. We always have vegetables with meals, that kind of things. (U5)

This situation caused sadness, and one of them expressed emotionally:

I have done exactly the same as everyone else (sobbing, tears falling). I have not noticed any difference to the others, I do the same as they do. (U4)

Participants also described “comfort eating”. Those who attributed their obesity to genetics and heredity struggled to find a correlation with their lifestyle habits. They found the situation unfair and were very emotional when speaking about their situation and their attempts to resolve it.

#### Challenges in changing behaviour

All of the adolescents focused on behavioural changes; feeling highly responsible and coming up with suggestions for what to do. They shared experiences from multiple attempts to lose weight, describing challenges, including maintaining good eating habits during daily life and holidays, eating less energy-dense food, and the risk of succumbing to temptations. The culture influenced their choice of food; however, they made efforts to moderate their intake. One adolescent put it in this way:

I usually eat what I like, but I could, maybe, eat smaller portion sizes. (U1)

One of the girls told us how she also struggled to influence the whole family’s eating habits, including those of her siblings.

It is scary for me to observe my brother and the twins becoming bigger. I tell the twins they must eat less, if not they will end up as me. Yesterday I told my brother he should eat breakfast and not skip meals, because otherwise he will eat huge portion sizes. I do not know if he listens to me or shuts his ears, but anyhow, I gave him my advices. (U4)

Physical activity was a recognized need but was challenging for most of them, illustrated in this quote:

I’m aware of my obesity. I’m in poor physical shape and I get tired more easily than my peers in my football team. Being active takes more out of me than them. (U1)

Further, social support was considered important in terms of performing physical activity. This participant put it in this way:

I know a lot about training. A friend of mine exercises often, and I have learned a lot from him. (U1)

### Bullying and fragile social relationships

All of the adolescents emphasized social relationships and stated that friends, peers and families were particularly important in terms of the interviewees’ struggle with obesity. They felt vulnerable because they were “different” and expressed a strong wish to be similar to others. Both social support, as well as lack of integration, strongly influenced everyday life and challenges living with obesity.

#### The important, but deceitful friends

The interviewees spontaneously brought up the subject of friends, and friendship obviously meant a lot to them. In response to one of the researcher’s exploring questions about friends, one of the youngsters said:

Yes, I do have friends (talking quietly, tears falling). (U2)

They expressed the view that having the support and respect of friends was essential. Having friends was important for emotional support and motivation for joining physical activities, as illustrated by one participant:

I felt that I did not have many friends there, so I stopped joining. It’s not nice when you do not have anyone similar to that: friends, to be with. (U3)

This quote, on the other hand, shows how activity and friendship are connected:

It is more fun playing football, because I have friends there. (U1)

However, their existing social relationships seemed fragile. Unfortunately, some experienced betrayal by friends who tried to sabotage their attempts at behaviour changes. One said:

If I sleep over, and I’m trying not to eat so much candy, she says: “Take another one!” She does this just to irritate me. (U3)

The participants feared being discredited in social settings generally, and in particular during physical activities. They experienced lack of social support in situations involving organized sports, and all but one had experienced difficult situations relating to their weight and appearance. Some experienced bullying, including being insulted, experiences with a lasting effect on their emotional state and motivation to make behavioural changes. They then felt sad and found it hard to remain positive about the future.

The school setting was a highly important social arena, and the interviewees expressed both a need for, and a lack of, social support. One of the girls described how she was afraid to speak in school settings in case she said something wrong. She feared her schoolmates might point at her, draw attention to her and make her feel stigmatized. She put it in this way:

I was called fat during six or seven years during elementary school, and for that reason I tried to become invisible. I became more shy. (U5)

Among the girls, being insulted by the boys was common. This quote makes an illustration:

I think it was in 4th or 5th grade; a boy in my class said something to me, like: “Did you eat much breakfast today?” And one of my classmates used to say that my girlfriend and I were fat. Then, later, in 6th grade, two boys in my class said to me: “You’re ugly.” I think it was in the 4^th^ grade the boys’ insults got worse. I was called Fat Doris (not her real name) and “The fat one”. I used my weight as a weapon when they were rude to me. I tried to scare them and said I would sit on them. However, in the 6th and 7th grade I could not bear it anymore. I was completely defeated. The bullying has made it difficult for me to lose weight. I have, you know, gained weight because feeling that I was being bullied has hurt me inside. (U4)

The same girl had a deceitful boyfriend:

He behaved okay towards me when we were alone, but (taking a deep breath) when he was with his friends, he was not kind to me at all. (U4)

Ending or mending these unsupportive friendships proved hurtful and hard to handle, and sometimes help was needed, as described in the following quote:

I have had some problems with my friends. I had real conflict with my best friend, and talking to the school nurse helped me a lot in solving it. (U4)

Often, only a few friends were trusted partners in conversations about the adolescents’ obesity-related challenges. One girl thought it would be helpful to meet with peers also suffering from obesity in a group:

It would be nice to know how other adolescents feel about it, if they have been bullied and so on… (U2)

#### The family as the primary source of support

For the adolescents interviewed, parents as well as extended family seemed to be of great importance in terms with living with obesity. The adolescents shared several positive experiences and expected their families to help them solve the situation:

I do not think many obese children have family members such as me, who intervened and made contact on my behalf. I’m so grateful that she (the partner of the grandfather) did. (U2)

My parents set limits to what I should eat, and when we are on holidays they tell me what kind of food I should choose. (U1)

One said:

Just to have someone to talk to would be of great help. (U5)

Some of the adolescents expressed a wish for more extended familial support. One of the adolescents described this as follows:

I wish I could get the money to join a fitness centre. That is what I have told Mom and Dad, and that is what I want: to go there for a whole year. Then, I could exercise two or maybe three times a week, and there they also give you advice on how to exercise. (U3)

Unfortunately, for some, their expectations of support were not fulfilled, as illustrated by this quote:

My mom does not have a problem with her weight, and she does not know much about obesity. (U4)

In such situations, they sought other sources for support:

When I told my mom and dad that I was being bullied and beaten up at school, they did not believe me. My aunt and grandma did; however, they were unable to stop the bullying. (U4)

Several participants pointed to how the extended family could be an asset for them:

My grandma and grandpa have been a great help to me. My grandpa says: “Now you have reduced your weight!” He praises me for what I have achieved. In addition, grandma also knows about my problem as well as my aunt, and they give me some advice, because my grandma works at a nursing home, she knows about food and that kind of thing. (U4)

I have received considerable help and support from my grandfather(starts crying). (U5)

Thus, the extended family in varied ways became valuable sources of support.

### Obesity as a shameful and sensitive issue

A prominent phenomenon during these interviews turned out to be the shame that the young people appeared to feel about their condition and the difficulty in talking about obesity-related issues with other people. At the same time, the participants made clear statements about their need to talk about their weight problem, despite difficulties in verbalizing their struggles. They expressed a wish that other people take their weight problem more serious, and they were troubled by the way in which their condition was sometimes talked about.

#### An urge for secrecy

Several adolescents tried to keep their awareness of their own obesity and the challenges they faced secret. Those who felt it was useless to try changing their body weight, because they considered their condition “genetic”, described the condition as being difficult to talk about, an issue that made them feel vulnerable in most settings. This was described in different interviews with the adolescents:

I do not talkmuchwithfriends about my being obese. (U3)

I amembarrassed,and I do not dare reveal how it feels to be bigger than the others […] I do not want them to know that I feel bigger than them. I want this to be private. (U2)

I have not participated in swimming classes this year, neither last year. I do not like to go with—(mutters) this size of mine. I feel people should not see me like that. (U5)

A participant who suggested being in a group for obese adolescents would be helpful stated:

I would not like people to know I was in a group such as that. Other people do not need to know I have joined that kind of group. (U2)

Those subjects who avoided telling others about their troubles and their attempts to lose weight reported feelings of sadness and hopelessness.

#### The ambiguity of asking for help

The participants expressed differing opinions about seeking help. Recognizing the need for help, and asking for help, appeared challenging. Asking for help would threaten their secrecy. This was described as follows:

I do not think many people contact the health care service about obesity-related problems. […] I do not want to involve health-care professionals. I do not want the school to know about it. (U2)

Those who observed their obesity as inherited also doubted in what way the health-care services could support them:

I do not know what I would such as the health care provider to say or do. I do not know at all (whispers). (U2)

I have not searched for help from any healthcare service. I do not know (laughing). I was near to say: That is what my mom is doing, trying to find what would be suitable for me. (U5)

Hence, society’s perceptions of obesity and its causes prevented some of them from asking for help.

#### The complex health-care encounters

The participants had experienced encounters with general practitioners (GP), school health nurses and physiotherapists. The participants were grateful when someone did take action and offered help. At the same time, the outcome of the encounters differed. Their ambivalence towards the encounters was clearly expressed:

If the school health nurse had talked about obesity when she measured me, it would be OK for me to talk with her, but I would never go back to talk more with her about it. (U3)

In cases where support was offered, it was received with gratitude, as this informant said:

The school health nurse asked me how I had been doing since last time I met her. Now I see her every two weeks. […] I am grateful that I have been able to get help, I think they offer as much help as they can, to support me. […] The doctor (GP) contacted a training centre for adults. He tried to ask for the same service for adolescents, since there are many obese adolescents in my community; however, we received a letter saying that it would ‘take a while’. (U4)

Other encounters offered opportunities to find a cause for the person’s obesity, which was a solution that was not always found to be helpful, as illustrated in this quote:

I do not remember what the doctor (GP) said to me, but he took some blood tests to discover if the problem was low metabolism, or inherited from my father. However, there was no connection. I do not think he (the GP) did anything else […] he might have mentioned the school health nurse. (U3)

Assistance and encouragement from a physiotherapist was beneficial:

I go to the physiotherapist twice a week to exercise [….] and they have given me considerable praise […] I have felt better after exercising. (U4)

Further, the obese adolescents had a strong opinion on aspects of the support that they needed. It seemed important that the helpers did not go too quickly when dealing with the issue of obesity, demand too much from them, or use certain words. One adolescent said:

When I speak with the school health nurse, she does not tell me that I have to diet, but she tells me I should keep the current weight and kind of reduce some grams and kilos. You see, she never mentions *dieting*. Because if she does, then I hear *slim*, and I do not want to be slim, to make changes in a hurry, and for everything to be done as quickly as […] I do not want them to demand too much, but to help me to reach my own goals. (U4)

The helper should not demand too much weight loss because then the adolescent feared that he would not succeed, and the burden of shame, responsibility and loss of support could increase.

## Discussion

The aim of the study was to gain an in-depth understanding into the perspectives and life experiences of adolescents living with obesity. Our analysis revealed how these youths experienced a daily struggle to find ways to lose weight. Most of them felt unsafe in social relationships, were stigmatized and bullied and were unsure if anyone could help them. Ambivalence for help seeking was prominent. Ideally, they wanted help in the family. The youths who came from families with obesity experienced distress, shame, sadness and feelings of hopelessness, and some of them had given in.

Adolescents interviewed in this project perceived obesity and causes for obesity differently. Perceptions ranged from the simple explanation of *eating too much and moving too little* to the fact that most individuals in their families suffered from genes that promoted obesity. This focus on biological determinants and genes among adolescents has seldom been described. In contrast, Nguyen and colleagues (2017) described adolescents with obesity who stated that eating and lack of physical activity caused their obesity. A meta-synthesis of qualitative studies found that adolescents had difficulty in perceiving and labelling obesity (Lachal, Orri, Speranza, 2013). The fact that genes influence the risk for obesity (Campión, Milagro, & Martínez, 2010) was known to the adolescents in this study, a knowledge that made some of them feel trapped, thinking that the situation was out of control. Current assumptions highlighted the importance of personal responsibility as a prerequisite for self-determined action against obesity (Braun et al., 2014). Our study describes how the adolescents who had parents with obesity seemed to suffer more and were less likely to receive familial support in making lifestyle changes. Consequently, they seemed to face greater challenges in remaining hopeful about the potential for a better life.

In our study, adolescents who stated that they ate the same diet and exercised as much as others perceived their obesity as being unfair and hard to live with. They could not understand why they were obese, and the situation made no sense to them. It is known that this group of adolescents experiences barriers to change and lack of motivation and perceives unfavourable comparisons with other adolescents (Smith, Kerr, Fenner and Straker, 2014). The adolescents in the study reported here described their struggles to resolve the situation, including difficulties in successfully decreasing body weight, a result that was also found by Kirk et al. (2014). These experiences add to our understanding of their challenges in achieving weight-loss and their need for support, corresponding to Pretlow’s findings in a web chat for adolescents with obesity, in which their cries for help were obvious (2009). Some of the participants seemed to have *given in*, preferring to internalize their feelings. Internalizing the sad feelings and suffering that obesity often involves might decrease the options open to the adolescents in terms of resolving their situation.

The participants stressed the importance of having friends who provided vital support for successful weight loss. The data describe the importance of close, reliable friends whom sufferers could depend on to help them. This emphasis on social support corresponds to the studies published by Braun et al. (2014) and Trogdon et al. (2008). Unfortunately, most of the interviewees in the study reported here felt unsecure and described a lack of support in many social settings, particularly at school, and they feared being pointed out as different and stigmatized. They expressed sadness about not being included in their school peer group, and they had a strong need to be similar to others. Adolescents living with obesity experience and perceive unfavourable comparisons with other adolescents (Smith, Kerr, Fenner; Straker, 2014). The participants in our study suggested that being in a group with other adolescents suffering from obesity was a preferred arena to share experiences, obtain peer support and perhaps participate in pleasant activities together. Most of them clearly expressed their lack of knowledge and support from friends and family. They appeared to be vulnerable and seemed to have fewer resources to handle teasing, bullying and stigmatizing or to succeed in health-promoting activities.

In this study, all of the interviewees had been bullied, except for one. Some stories reflected particularly damaging situations, and the bullied adolescents clearly expressed their suffering. Thomas and colleagues (2008) also found that some adolescents with obesity experienced stigmatization and discrimination. The girls in our study also expressed low self-confidence and self-esteem, and most of the interviewees expressed shame and self-blame. Shame and self-blame seemed to increase their hopelessness and was viewed as a barrier to the adolescents’ asking for help and making lifestyle changes. Adolescents’ feelings of responsibility for their weight status, guilt and shame were also found by Kirk and colleagues (2014)and Rees et al. (2011).

The family and the extended family seemed to be of great importance for the interviewees, as reported in other studies (Braun et al., 2014; Schalkwijk et al., 2015). Several of the adolescents interviewed in our study reported having obese parents. Even so, some of the adolescents expressed that their parents lacked the necessary knowledge or resources to be significant helpers. The adolescents stated, with emotion, that obesity should be taken seriously and spoken about, that they wanted help, and that they were unsure what to expect from health-care personnel. At the same time, corresponding to the adolescents in Thomas et al.’s study (2008), they emphasized the way in which the issue was brought to the surface. They revealed ambivalence; some subjects had a strong desire to keep their struggle hidden, without involving others, while at the same time revealing a desire for help. Those who considered obesity to be a genetic legacy expressed shame and hopelessness. These phenomena became barriers to seeking help. Shame, blame and guilt have been more specifically linked to the failure of these persons to achieve weight loss and to vulnerability, by virtue of being in need of help (Morinder, Biguet, Mattson, Marcus, & Larsson, 2011). Individuals living with obesity described their experiences in seeking support from a system that they believed was failing them, and when there was no clear reason for their unsuccessful weight loss, they chose self-blame (Kirk et al., 2014). Several adolescents in our study expressed the fear of being exposed to the challenge of making behavioural changes and the fear of failure in their attempts to lose weight. Failure, in this context, appeared to manifest as a feeling of being out of control, and they perceived such situations as involving vulnerability and shame. Ambivalence was also prominent. Ambivalence can originate through uncertainty and fear about whether they will be able to achieve what is required of them.

### Methodological considerations

The trustworthiness of qualitative studies can be evaluated within the concepts of credibility, dependability and transferability (Graneheim & Lundman, 2004: Rowan & Huston, 1997). *Credibility* addresses how well the data and analysis meet the study’s aim, the selection of meaning units and the abstraction of data through codes and categories to themes. We claim that the data in this study are rich and meet the aim of the study and the research questions. However, a weakness might be the challenges that the interviewees faced in terms of putting their experiences and worries into words. Adolescents may have diminished critical thinking, as well as trouble communicating own behaviour, meanings and emotions because of neurophysiological processes (Jensen & Nutt, 2016). Further, we performed a collaborative analysis with abstraction of data in reflexive dialogues and in several steps, aided by the QRS Nvivo to ensure credibility. During this process, illustrated in Table II, we challenged our pre-understandings and interpretations in constant dialogues. *Dependability* should take into account both data instability and changes induced by the design of the study (Graneheim & Lundman, 2004). In this study, the repeated interviews, and the same interviewers, reinforced the trustworthiness. During the second interview, the participants’ confirmed their main perspectives and experiences. However, we are aware that recall bias might have led them to agree when actually they could not really remember what occurred during the first interview. Concerning *transferability*, the participants were recruited from a small geographic area, the number of respondents was low, and the gender distribution was uneven, all of which represent limitations for transferability to other contexts. Nevertheless, we think that the rich data seem to represent universal life experiences that are essential for health-care personnel to be aware of to strengthen the patient-clinician partnership.

## Implications for practice

This study describes the importance of taking into account the adolescents’ perspectives, experiences and individual needs. First, we think that health-care providers should assess the adolescents’ thoughts on causal explanations of their obesity. We agree with Braun and colleagues, who claim that more focus needs to be directed to the interplay between the medical and social aspects of obesity, and that preventive strategies should include an awareness of the relevance of subjective ethnology when defining reasonable and effective governance strategies in tackling obesity (Braun et al., 2014). Furthermore, the adolescents’ ambivalence and psychosocial challenges should be focused on through the health-care encounters. Exploration of the relationship between bullying and obesity is needed along with the role of families and the home environment. With regard to mental health and learning in young people living with obesity, the school setting seems important, particularly if there is a focus on stopping teasing, bullying and stigmatization. To overcome barriers to healthy eating and physical activity in adolescents, stakeholders emphasize issues of stigma and social acceptance (Goh et al., 2009). There is a need for specialized expertise in schools as well as in the health-care services to ease the burden of these persons. Health-care professionals should also be aware that parents with obesity, who throughout their own life have struggled to address obesity, might have the same abusive experiences as the interviewees in this study describe and thus may lack the resources needed to support and assist their child with obesity. Thus, in these cases, the health-care providers support is even more important. We take the view that when adolescents with obesity have the courage to seek help, we must ensure that they encounter professionals with expertise in patient-centred communication who understand their situation and who show them respect. A crucial element is that shame and self-blame can act as a “resource thief” and be a barrier to seeking help. Further research should assess and identify the active ingredients within treatment programmes, which can help young people to manage their weight in the long-term (Reece, Bissell, & Copeland, 2016).

## Conclusions

Adolescents living with obesity described everyday life as difficult to interpret and solve. They perceived causes for obesity differently, and those who emphasized familial determinants seemed to face greater challenges. Lack of support from parents, trusted friends and health-care providers, as well as bullying, shame, guilt and self-blame, represented threats that decreased motivation for help-seeking and success in their repeated and varied attempts to change lifestyles. The adolescents were ambivalent towards disclosing their concerns and seeking help. Disclosure of their concerns seemed easier in groups of peers. Health-care encounters were experienced differently. It was emphasized that health-care providers should not demand too many and too quick behavioural changes and should avoid certain words, such as slimming. Health-care providers need to be skilled in assessing each individual’s resources and interpretations of their condition, to be able to communicate in a respectful, patient-centred manner and to assist each adolescent to explore their ambivalence and set their own realistic goals. We need more research to explore communication in encounters between health-care providers and adolescents living with obesity to develop and implement effective interventions. Young people living with obesity should be involved in the design of future interventions.
